# Compositional Diversity and Functional Interactions of Bioactive Compounds in Japanese Green Tea: Focus on Matcha, Sencha, and Gyokuro

**DOI:** 10.3390/molecules31162934

**Published:** 2026-08-21

**Authors:** Keiko Unno, Yoko Kameoka, Yoriyuki Nakamura

**Affiliations:** Tea Science Center, University of Shizuoka, 52-1 Yada, Suruga-ku, Shizuoka 422-8526, Japan; unno@u-shizuoka-ken.ac.jp (K.U.); gp1921@u-shizuoka-ken.ac.jp (Y.K.)

**Keywords:** green tea, matcha, sencha, gyokuro, functionality, compositional differences, stress modulation

## Abstract

Japanese green teas, including matcha, sencha, and gyokuro, are derived from the leaves of *Camellia sinensis* and contain bioactive compounds such as catechins, caffeine, and amino acids, notably theanine. However, the amounts and ratios of these components vary significantly depending on the harvest season, cultivar, and cultivation practices. Matcha products on the market range widely in quality, from high-grade ceremonial products to lower-quality powders. In sencha, compositional profiles differ not only according to harvest timing but also according to infusion conditions such as water temperature and steeping time. Gyokuro, a premium shaded tea, is a type of green tea with a particularly high theanine content, just like authentic matcha. The compositional differences are expected to influence the physiological and psychological functions of green tea. For instance, the stress-relieving effects of theanine are modulated not only by its concentration but also by its interactions with coexisting compounds such as caffeine and catechins. Similarly, the bioavailability and physiological activities of catechins may be altered by coexisting theanine and caffeine. This narrative review focuses on compositional variability and interactions among major bioactive compounds in Japanese green tea and discusses how these factors may influence their functional properties.

## 1. Introduction

Green tea is produced from the leaves of the *Camellia sinensis* plant. Immediately after harvesting, the leaves are subjected to steaming (Japanese method) or pan-firing (Chinese method) to deactivate oxidative enzymes [1], thereby preserving their natural green color. This processing step distinguishes green tea from fermented teas, such as black and oolong tea, which are derived from the same plant species.

Japanese green tea encompasses several varieties, including sencha and gyokuro, in which aqueous infusions of the leaves are consumed, as well as matcha, in which finely powdered leaves are ingested in their entirety [1]. Notably, gyokuro and matcha are produced from young shoots that have been shaded for at least 20 days prior to harvest. This shading process results in elevated amino acid content and reduced catechin levels, imparting a pronounced umami taste with minimal bitterness or astringency. In addition, shading induces the development of a distinctive aroma often described as seaweed-like. In contrast, sencha is produced from leaves cultivated under full sunlight and is characterized by a fresh aroma and a balanced profile of astringency and umami.

In recent years, matcha has gained global popularity, and it is often assumed that any powdered tea qualifies as matcha; however, this is not the case. Authentic matcha is produced from first-flush (the first harvest of tea leaves) tea leaves that have been shaded for more than 20 days prior to harvest. In contrast, so-called “food-grade matcha,” commonly used in confectionery and beverages, is frequently marketed as matcha despite being produced from lower-quality leaves that are insufficiently shaded or derived from later harvests [2]. “Powdered tea” refers to finely ground sencha, so while it is in powder form, it is not matcha. While these green teas differ in their contents of constituent components, the most notable difference is their theanine content. Given the increasing global demand for matcha, it is important to disseminate accurate information regarding quality distinctions. Types of Japanese green tea are shown in Table 1.

Extensive research in Japan has examined variations in chemical composition associated with matcha quality. Studies have identified total nitrogen and free amino acid content as key determinants of green tea quality, including both matcha and sencha [3,4,5]. Furthermore, compositional differences have been reported among authentic matcha, processed matcha, and powdered teas marketed as matcha [6]. Nevertheless, such quality-related information is not always adequately communicated in international markets. Additional complicating factors include price distortions due to tariffs and the decoupling of demand from quality. Despite these challenges, growing global interest has prompted increasing research activity on matcha quality outside Japan [7,8,9,10,11,12]. 

Variations in matcha quality arise from differences in chemical composition, which influence both taste and functional properties. This review summarizes analytical data on catechins, caffeine, and amino acids—key constituents of both matcha and sencha—to clarify their roles in determining quality. For example, theanine has been reported to exert stress-reducing effects [13]; however, its concentration varies substantially among green teas. Moreover, interactions among tea components are critical. Caffeine and epigallocatechin gallate (EGCG) may counteract the effects of theanine, whereas arginine exhibits similar functional properties [14]. Arginine is one of the most abundant amino acids after theanine in Japanese green tea. The balance among these compounds is thus of central importance. Nevertheless, we would like to state that there are very few studies that have comprehensively measured the differences in the content of various components between green tea and matcha and that some of our conclusions are based on our previously published research.

In addition to Japan, green tea made from *Camellia sinensis*—the same plant used in Japan—is produced in countries such as China, South Korea, and Vietnam. However, due to differences in growing conditions, production methods, tea varieties, and processing techniques, each of these countries produces green tea with its own distinctive characteristics. It is natural to assume, therefore, that green teas from other countries, while containing the same major components, have different effects due to differences in composition. However, such differences have not yet been scientifically clarified. 

This study does not provide a comprehensive evaluation of these various green teas; rather, it focuses on the compositional differences and interactions within Japanese green tea. Through our research on Japanese green tea, we demonstrate that even green teas containing the same components can exhibit different functional properties due to differences in their composition. We propose that our data on composition and interactions could serve as a reference for inferring health benefits in green teas from various countries, including Japan.

Furthermore, while numerous studies have investigated individual components such as catechins [15,16,17], caffeine [18], and theanine [19], as well as epidemiological and clinical effects of green tea [20,21] and matcha [22,23,24,25], a comprehensive understanding of green tea functionality requires consideration of interactions among these components. This narrative review does not aim to comprehensively and systematically evaluate all reported health effects of green tea; rather, its purpose is to discuss how differences in components, cultivation, processing, and extraction conditions may influence physiological functions. This research thus focuses on the variability of components in Japanese green tea—particularly matcha, sencha, and gyokuro—and the interactions among major bioactive compounds. In addition, it focuses on studies examining the interactions between theanine and EGCG, theanine and caffeine, EGCG and caffeine, and other related components.

It should be noted that some mechanistic and clinical studies discussed in this review were conducted using purified compounds, green tea extracts, or non-Japanese green tea materials. The findings may not therefore fully reflect the physiological effects of authentic Japanese green teas, whose chemical compositions vary substantially depending on cultivar, shading practice, harvest season, and preparation method.

## 2. Matcha

In matcha production, green tea plants are shaded for over 20 days prior to harvest. Under direct sunlight, theanine—one of the primary umami components in tea leaves—is rapidly metabolized into glutamic acid and ethylamine, with the carbon from ethylamine primarily converted into catechins. In other words, shading inhibits the degradation of theanine and suppresses catechin synthesis, which is responsible for astringency [6]. Shading also increases chlorophyll content, resulting in a deeper green coloration [26]. Traditionally, materials such as reed blinds and straw mats are installed on shelf-like structures in tea fields to block direct sunlight [27]. This approach facilitates adequate airflow and contributes to high-quality green tea production [28]. In recent years, however, methods employing shade cloth or sheets placed directly over the tea plants have become increasingly common [29]. When sufficient shading is applied prior to harvest, matcha exhibits a deep green color, reflecting elevated chlorophyll levels [30]. Furthermore, when first-flush tea with high theanine content is used and catechin synthesis is adequately suppressed through shading [31], the resulting green tea exhibits reduced astringency.

Tea leaves cultivated under shaded conditions are steamed immediately after harvesting and then rapidly dried using high-temperature air in a specialized device known as a “tencha dryer,” without undergoing rolling. The resulting product is referred to as tencha. Matcha is produced by grinding tencha into a fine powder using a stone mill or mechanical grinder [2]. However, due to the prohibitive cost of tencha dryers, tea leaves dried using equipment designed for sencha production are also widely utilized [2]. Although these leaves are similarly dried without rolling, they differ from authentic tencha in several important respects. When dried at high temperatures in a tencha dryer, matcha develops a characteristic sweet, roasted aroma. In contrast, the temperatures in sencha dryers are insufficient to produce this aroma, and the leaves may instead develop a blackish coloration. Additionally, the leaves may become slightly damaged or twisted during the drying process. When such leaves are ground into powder, the removal of hard petioles and leaf veins becomes more difficult. In the production of conventional tencha, heavier stems, petioles, and leaf veins are removed through sifting and discarded. Consequently, tea leaves produced using these simplified methods are typically used as raw materials for processed matcha [2].

Matcha produced from first-flush tea leaves contains the highest concentrations of amino acids and caffeine accumulated during the winter season, resulting in high-quality matcha rich in theanine and caffeine [6,28]. In contrast, the use of second-flush tea leaves or leaves harvested in autumn for matcha production has increased in recent years [2]. These leaves grow rapidly, leading to a lag in the supply of theanine from the roots relative to demand, while elevated temperatures accelerate theanine metabolism. Furthermore, these leaves are subjected to shorter shading periods or minimal shading, resulting in significantly lower theanine content. Caffeine levels also decline after summer. Conversely, catechin content reaches its peak during summer and is stored in cellular vacuoles with minimal metabolic degradation occurring [32]. As a result, these tea leaves—characterized by low theanine and high catechin content—are primarily used as raw materials for processed matcha.

### Differences in the Composition of Matcha Sold in Japan and Abroad

**Figure 1:**
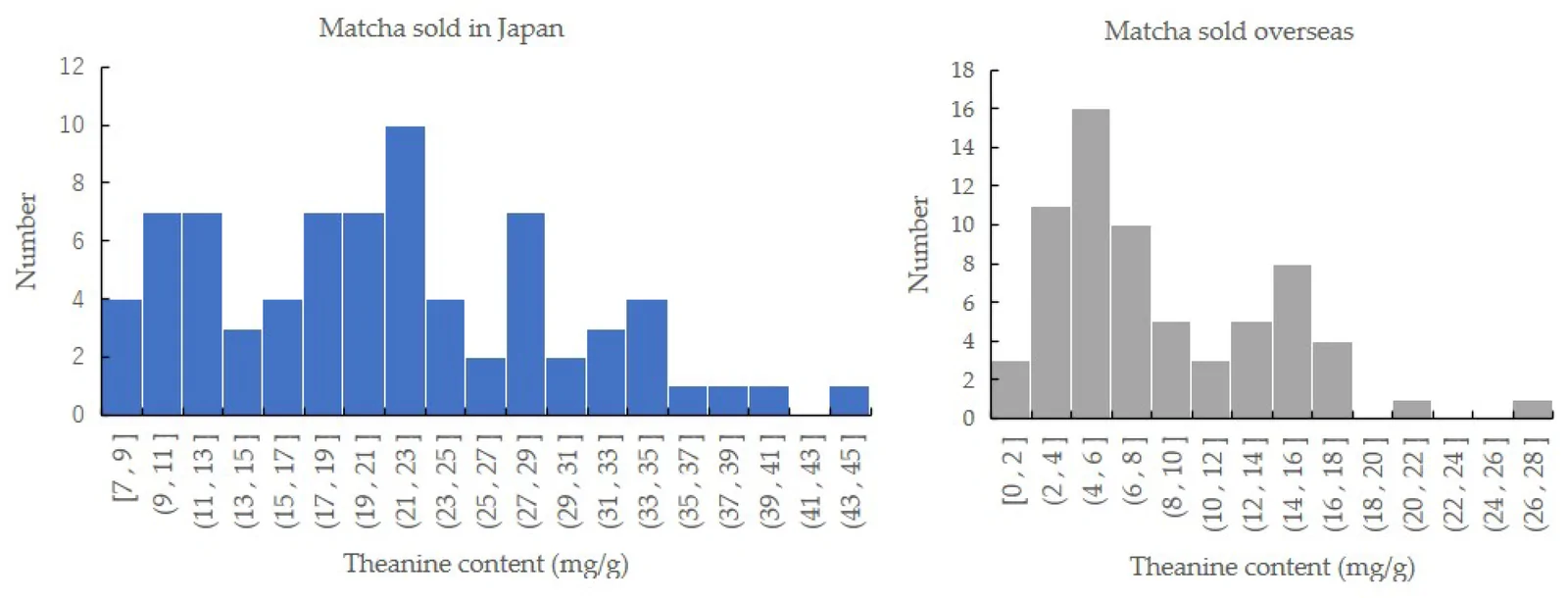
Distributions of theanine content in the analyzed matcha samples. The number of matcha samples corresponding to each theanine content level (horizontal axis) is plotted as a histogram on the vertical axis. These results are based on a reanalysis of data published in Reference [33] regarding 75 matcha samples sold in Japan and 67 matcha samples sold overseas in 2017. For this reanalysis, one sample of sweetened matcha sold in Japan was excluded, resulting in a total of 75 samples.

The distributions of theanine content in the analyzed matcha samples are represented as histograms (Figure 1). For matcha sold in Japan, the three samples with the highest theanine content were classified as “higher,” and the three samples with the lowest content were classified as “lower.” For matcha sold overseas, the three samples corresponding to the median were classified as “medium” theanine content.

Table 2 presents the mean values for three high- and three low-theanine samples selected from matcha brands sold in Japan, as shown in Figure 1. High-theanine matcha contained 41 mg of theanine per gram of tea, whereas low-theanine matcha only contained approximately 20% of that concentration (8.5 mg). Similarly, levels of other amino acids were also reduced. The caffeine content in low-theanine matcha was approximately 80% of that found in high-theanine matcha. In contrast, EGCG levels in low-theanine matcha were 1.4 times higher than those in high-theanine matcha. Other catechins—epigallocatechin (EGC), epicatechin gallate (ECG), and epicatechin (EC)—also increased by 1.3- to 3.7-fold. 

Similarly, analyses of matcha ranging from ceremonial-grade to food-grade have reported that amino acids and caffeine decrease as grade decreases, whereas other metabolites such as polyphenols tend to increase [7]. These differences in quality indicate differences in their potential use as nutritional supplements.

An analysis of 67 matcha brands sold outside Japan revealed that although some products contained high levels of theanine, such products were extremely rare [33]. Table 2 shows the mean values for three brands that exhibited moderate theanine levels. These values were comparable to those of low-theanine matcha sold in Japan, suggesting that in 2017, matcha with even lower theanine levels was widely available overseas. In fact, matcha used overseas is primarily incorporated into processed foods such as confectionery. However, the fact that there are differences in the compositional components of matcha may not be widely known, and considering the recent expansion in the range of uses for matcha, new data on the types of matcha currently available on the market is thus needed.

Matcha has traditionally been considered a type of green tea rich in theanine due to its unique cultivation practices [28]. Accordingly, it is widely recognized as providing theanine-associated stress-reducing effects. However, it should be noted that matcha with low theanine content may not confer such benefits [33]. Commercially available matcha products typically lack labeling regarding theanine content, making it difficult for consumers to assess their potential stress-reducing effects based solely on packaging. Although price previously served as a useful indicator in Japan [28], recent increases in the cost of even processing-grade matcha have diminished its reliability as a benchmark.

Matcha with relatively low theanine content is commonly used as a processed ingredient in confections and beverages such as smoothies. In sweet products, catechin-rich matcha counteracts excessive sweetness through its characteristic bitterness while also enhancing visual appeal with its vibrant green color. In addition, numerous studies have demonstrated the health benefits of catechins, including their antioxidant properties [12], and their application in functional beverages has been proposed [34].

Furthermore, studies examining the nutritional composition of matcha and powdered tea used as food ingredients have shown that they are particularly rich in fat-soluble vitamins, including vitamin A (β-carotene equivalents), vitamin E (α-tocopherol equivalents), and vitamin K [35]. Accordingly, consumption of matcha alongside vegetables may contribute to improved intake of these nutrients. Matcha lipids are also characterized by a high content of unsaturated fatty acids, particularly alpha-linolenic acid, an *n*-3 fatty acid [36]. This compound is an essential fatty acid in humans and serves as a precursor for the biosynthesis of docosahexaenoic acid and eicosapentaenoic acid, both of which are associated with various health benefits [37].

In addition, matcha provides a moderate amount of dietary fiber [35,36] and may also be considered a source of plant-derived protein. Previous reports indicate that matcha contains approximately 17.3 g of protein per 100 g [36], while the Japanese Food Composition Tables report a value of 29.6 g per 100 g, comparable to that of soybeans (33.8 g per 100 g). Although matcha is typically consumed in relatively small amounts, it may still contribute to overall protein intake.

More recently, matcha has been found to contain substantial levels of microRNAs, with concentrations varying depending on cultivar and harvest season [38]. Given the increasing interest in the functional roles of plant-derived microRNAs, additional health-promoting properties of matcha may yet be identified.

## 3. Sencha

Sencha is produced from tea leaves grown in full sunlight. The manufacturing process involves steaming freshly plucked leaves to inactivate enzymes responsible for oxidation, followed by rolling and drying to form characteristic needle-shaped leaves [1]. In some cases, oxidation is instead halted by pan-firing, resulting in leaves with a more rounded shape.

The chemical composition of tea leaves varies depending on the timing of harvest [1]. Sencha produced from the first harvest is referred to as first-flush tea. The second crop is typically harvested 45 to 50 days after the first, generally from late May to mid-June. Tea harvested later in the season, typically from late September to late October, is known as autumn-flush tea. Compared with first-flush tea, autumn-flush tea contains significantly lower levels of theanine, slightly lower levels of caffeine, and higher levels of catechins. In addition, tea made from mature, hardened leaves rather than tender young shoots is classified as bancha, regardless of harvest timing.

First-flush tea leaves contain higher levels of amino acids than those harvested later in the season [1]. This is attributed to the accumulation of nutrients in the developing first-flush leaves, as well as the relatively slow growth rate of new shoots. Furthermore, even within first-flush tea, leaves harvested at a later stage tend to contain lower levels of theanine and caffeine and higher levels of epigallocatechin (EGC) and epicatechin (EC) [39].

Table 3 presents the chemical composition of different types of sencha. For the first-flush tea, 18 pieces of green tea were collected from various regions across Japan in 2022, and nine of them were selected, and these green teas were blended to create a test tea [40]. The second-flush samples were produced at the Mie Prefectural Agricultural Research Center for research purposes in 2023, as described in Ref. [41]. Similarly, the autumn-flush sencha samples were commercially purchased from a supermarket in Mie Prefecture in 2023 [41]. Previous studies have reported that the theanine content is approximately 20 mg/g in gyokuro, 10 mg/g in sencha, and 5 mg/g in bancha [4]. Accordingly, the first-flush sencha and gyokuro shown in Table 3 and Table 4 can be considered relatively high-quality based on their theanine content. Differences in chemical composition among these sencha samples suggest that their functional properties may also vary.

### Infusion of Sencha

Research based on sufficiently reliable scientific evidence has already been conducted regarding brewing methods for Japanese green tea [42,43,44]. In sencha, the composition of tea components in the infusion varies depending on the brewing conditions. Table 4 shows examples of component quantities for different sencha samples brewed under commonly used extraction conditions (leaf quantity, water temperature, steeping time, and water volume). For sencha harvested at the earliest stage, the picked leaves are softer; slightly cooler water at 70 °C was therefore used. For first-flush tea harvested at the middle stage, hotter water at 90 °C was used. Table 4 presents data for a 1 min infusion time. For second-flush tea and autumn-flush tea, results from steeping at a higher temperature (80 °C) for 1 min are also shown for comparison. Table 4 shows the average values based on data from three experiments for the first-flush tea. The data for the second-flush and autumn-flush teas are based on a single experiment.

**Figure 2:**
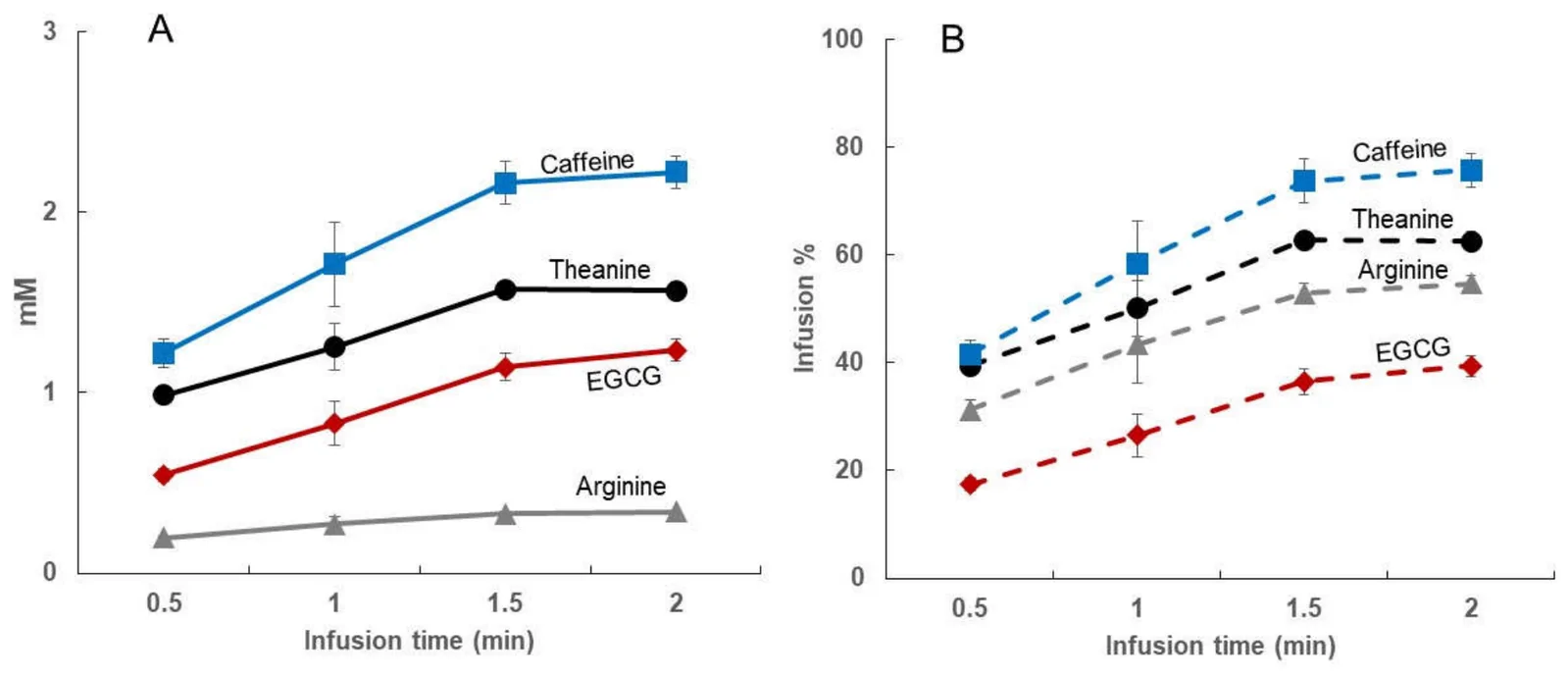
Increase in extraction of green tea components with steeping time. Concentration (mM) (**A**) and infusion (%) (**B**). Six grams of first-flush tea leaves (mid-harvest stage) were steeped in 260 mL of water at 90 °C for 0.5–2 min. Each point represents the mean ± SD (*n* = 3). These data are from a reanalysis of Reference [40].

Figure 2A shows that, when using first-flush tea (middle stage) and extending the brewing time, the amounts of infused components—theanine, arginine, caffeine, and EGCG—increase over time. The infused amounts increased rapidly up to 1.5 min but remained nearly constant between 1.5 and 2 min. Figure 2B shows the percentage of each component infused from the tea leaves. For caffeine, theanine, and arginine, more than 50% was infused after 1.5 min. In contrast, EGCG showed an infusion rate of approximately 30%. Sencha tea is generally brewed with water at 70–90 °C for 1–2 min. As shown in this figure, an infusion time of 2 min or less is appropriate when first steeping sencha.

**Figure 3:**
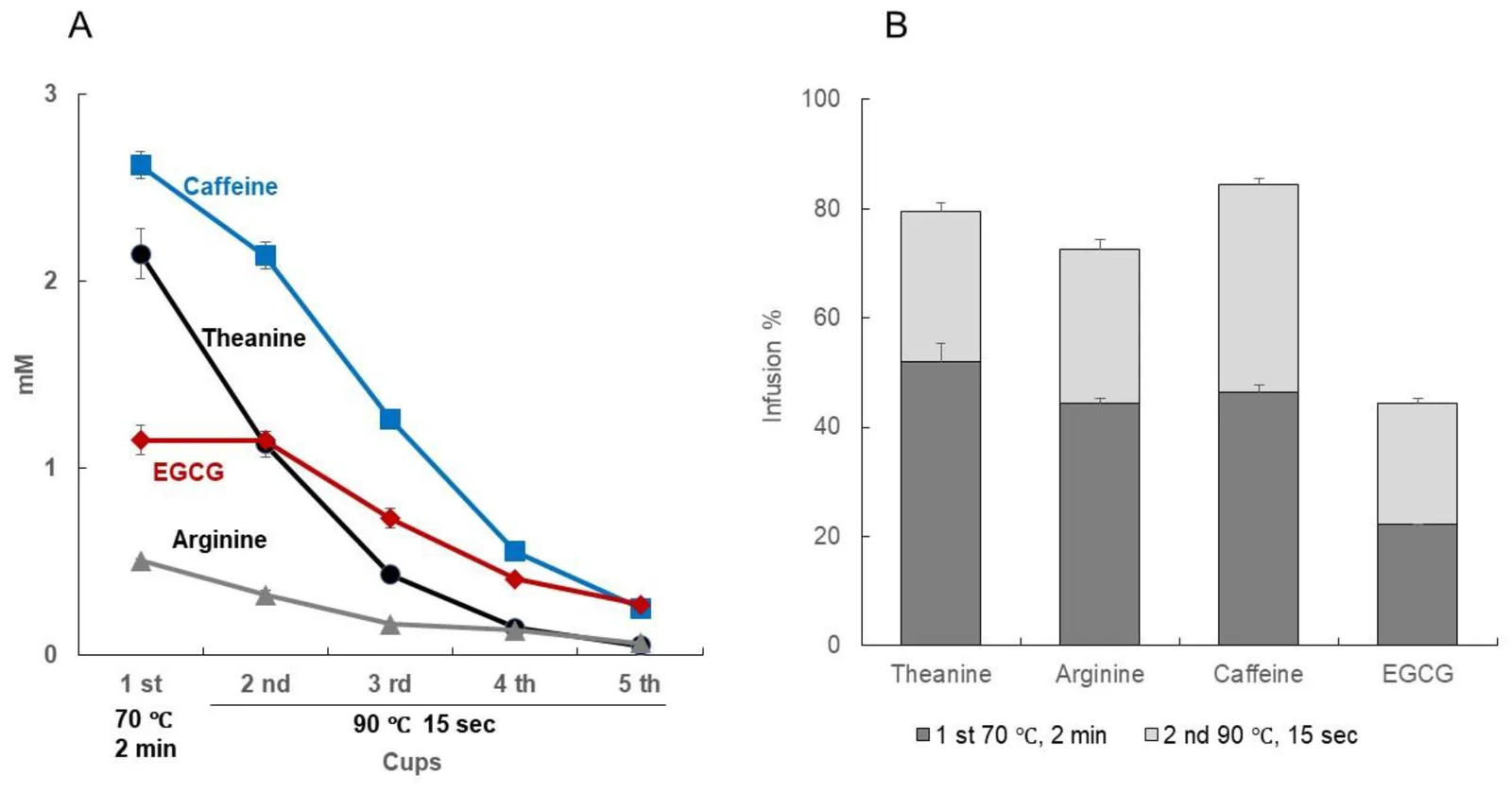
Green tea component extraction from the first to the fifth cup (**A**) and the elution rate for the first and second cups (**B**). First infusion: 6 g of first-flush tea leaves at the earliest stage were steeped in 170 mL of 70 °C water for 2 min (first cup). Next, 170 mL of 90 °C water was poured over the leaves and steeped for 15 s (second cup). The same process was repeated for the third through fifth cups. Each point and column represents the mean ± SD (*n* = 3). These data are from a reanalysis of Reference [40].

With sencha tea, the same leaves are steeped multiple times by adding hot water. The composition of tea components in the infusion was examined to determine how it changes in practice. When the first infusion was steeped for 2 min in water at 70 °C, the highest extraction rates were observed for caffeine, theanine, and arginine (Figure 3A). Subsequent infusions (second cup and beyond) were steeped for 15 s in water at 90 °C. Compared with the first cup, the infusion rates decreased significantly from the second cup onward. The infusion rate in the first infusion accounted for more than 50% of the total theanine content in the tea leaves and more than 40% of the arginine and caffeine (Figure 3B). While Figure 2 shows a higher infusion rate for caffeine than for theanine, in Figure 3B, the infusion rate for theanine is higher than that for caffeine because the water temperature for the first infusion was relatively low at 70 °C. Furthermore, by the second cup, approximately 80% of the total caffeine, theanine, and arginine contained in the tea leaves had been infused. In contrast, although the EGCG concentration was lower than that of theanine in the first cup, it became higher than that of theanine from the second cup onward. However, only 40% of the total EGCG contained in the tea leaves was infused by the second cup. While nearly all of the theanine, arginine, and caffeine contained in the tea leaves had been infused by the fifth cup, only about 70% of the EGCG had been infused.

The infusion data for green tea components presented here were obtained using Japanese tap water (soft water). Previous studies have shown that the infusion of catechins and caffeine decreases when hard water is used [45]. This effect is primarily attributed to the auto-oxidation of EGCG and ECG under alkaline conditions, followed by polymerization, which results in their degradation.

When preparing sencha, not only the amount of tea leaves, water temperature, water volume, and infusion time, but also the hardness of the water should therefore be carefully considered.

## 4. Gyokuro

Gyokuro, a premium form of sencha, is produced by shading tea plants from direct sunlight using reed screens or synthetic shade nets for approximately 20 days following the emergence of new shoots. This shading process suppresses catechin biosynthesis, thereby reducing astringency while enhancing the accumulation of umami-related compounds such as theanine. Consequently, gyokuro is characterized by higher theanine content and lower catechin levels compared with standard sencha (Table 5).

The gyokuro samples analyzed in this study were green teas produced in 2022 in major production regions of Japan, including Yame (Fukuoka), Uji (Kyoto), and Asahina (Shizuoka) [40]. Nine gyokuro teas were purchased from these areas, six of which were selected, and these teas were blended for test material. The data for each infused solution represent the average of three examples.

In general, gyokuro is brewed at relatively low temperatures (50–60 °C) for extended durations (2.0–2.5 min) to maximize the infusion of amino acids while minimizing the release of catechins and caffeine. In contrast to sencha, gyokuro preparation employs a higher leaf-to-water ratio, producing an infusion with exceptionally high concentrations of theanine and other amino acids. Although caffeine and catechins are also present at relatively high levels, their infusion is limited under these lower temperature conditions compared with that of theanine. A typical serving volume for gyokuro is approximately 20–30 mL per cup, and it is usually enjoyed in just a few cups.

**Figure 4:**
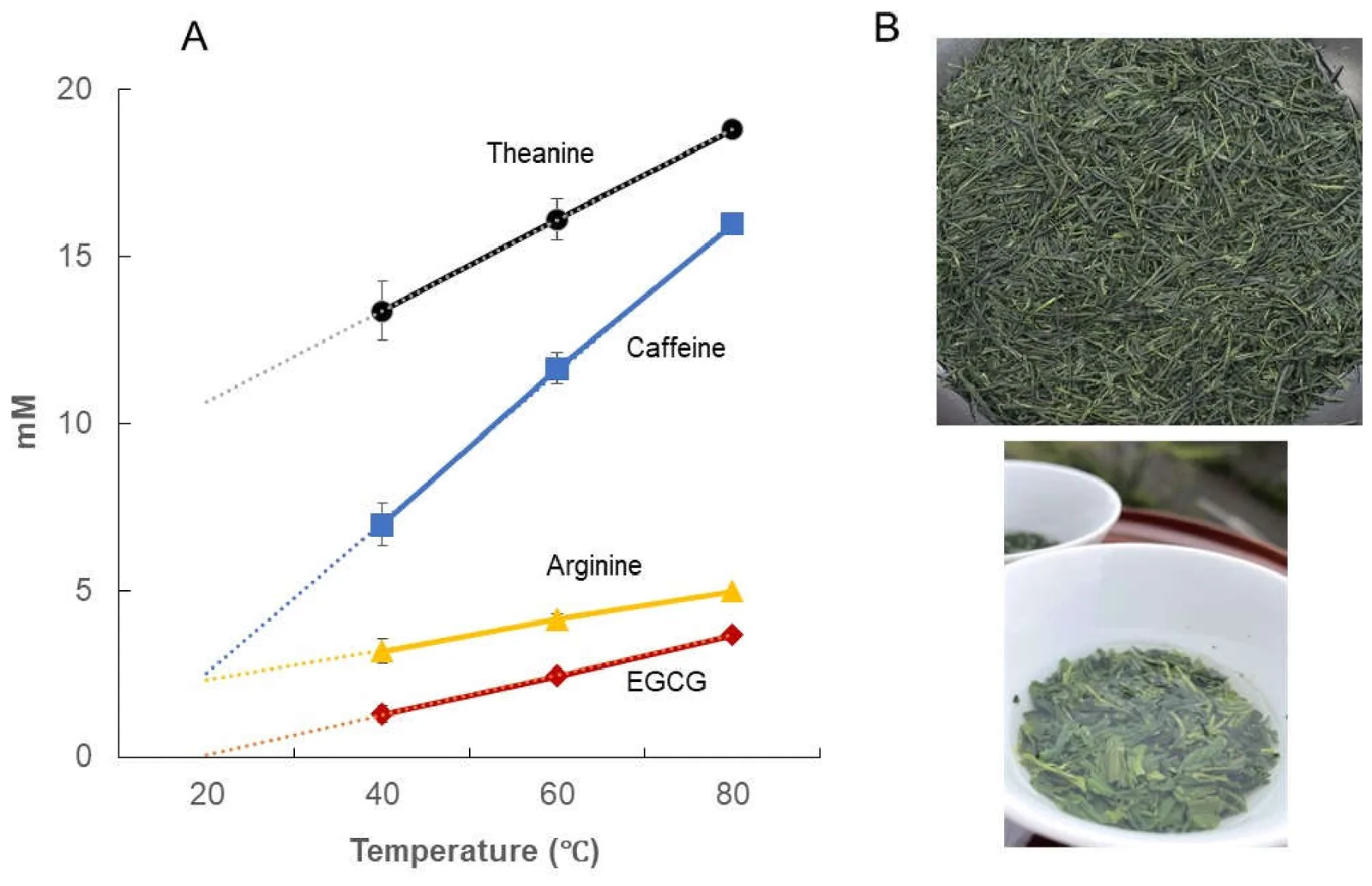
Changes in the concentrations of theanine, arginine, caffeine, and EGCG when gyokuro is brewed at 40–80 °C are shown in (**A**). A volume of 60 mL of water at each temperature was added to 10 g of gyokuro, which was then steeped for 2 min. The dashed lines indicate approximate trends. Each point represents the mean ± SD (*n* = 3). Photos of gyokuro tea leaves and *susuri-cha* (sipping tea) (**B**). An amount of 2 g of gyokuro was steeped in 30–40 mL of ice-cold water and left to stand for at least 10 min. This data is from a reanalysis of Reference [40].

Figure 4A illustrates the changes in the concentrations of theanine, arginine, caffeine, and EGCG in gyokuro infusions prepared at water temperatures ranging from 40 to 80 °C. Increasing water temperature results in higher concentrations of all components, with caffeine exhibiting particularly pronounced increases. These results indicate that infusion with cold or ice-cold water (approximately 20 °C or lower) markedly suppresses the extraction of caffeine and EGCG. In contrast, the concentrations of theanine and arginine are relatively insensitive to temperature, suggesting that cold-brewed gyokuro provides a beverage with enhanced umami and minimal bitterness or astringency.

For example, approximately 2 g of gyokuro leaves may be infused in 30–40 mL of ice-cold water for at least 10 min (Figure 4B). The clear supernatant is then gently sipped from the surface. This distinctive preparation method, unique to gyokuro, is known as *susuri-cha* (sipping tea). Furthermore, because gyokuro is made from soft, high-quality leaves, its infused tea leaves can be eaten after brewing.

It should be noted that some products are marketed as gyokuro despite undergoing shorter shading periods; such teas typically contain lower levels of theanine and higher concentrations of catechins, resulting in inferior quality.

## 5. Interactions Between Green Tea Components

This section presents recent findings on the functional properties of green tea components resulting from synergistic and antagonistic interactions and protective effects. PubMed was used for the literature search. Using the five keywords “EGCG,” “caffeine,” “theanine,” “arginine,” and “interaction,” we searched for reports published up to 2025 and identified 87 reports, including duplicates. From these, we selected studies that empirically verified various biological effects using cells, laboratory animals, or other models. Since this review focuses on interactions among green tea components, it includes not only basic research using purified compounds but also studies using green tea with clearly defined component concentrations. Furthermore, rather than limiting the scope to Japanese green tea, the review incorporates the results of meta-analyses and systematic reviews of epidemiological studies based on the perspective of interactions among green tea components.

### 5.1. The Relationship Between Theanine, Arginine, Caffeine, and EGCG: Stress-Reducing Effects

[Animal study] In experimental mouse models, stress levels can be assessed by the degree of adrenal hypertrophy [46], which reflects increased secretion of corticosterone (i.e., cortisol in humans). Although circulating corticosterone levels are commonly used as a stress marker, they exhibit circadian variation. In contrast, adrenal hypertrophy peaks one day after stress exposure and persists for at least one week [46]. Theanine and arginine have been shown to exert stress-reducing effects, whereas caffeine and EGCG exert antagonistic effects [14]. In addition, glutamic acid and aspartic acid antagonize the effects of theanine, and EGC counteracts the activity of EGCG [14]. 

**Figure 5:**
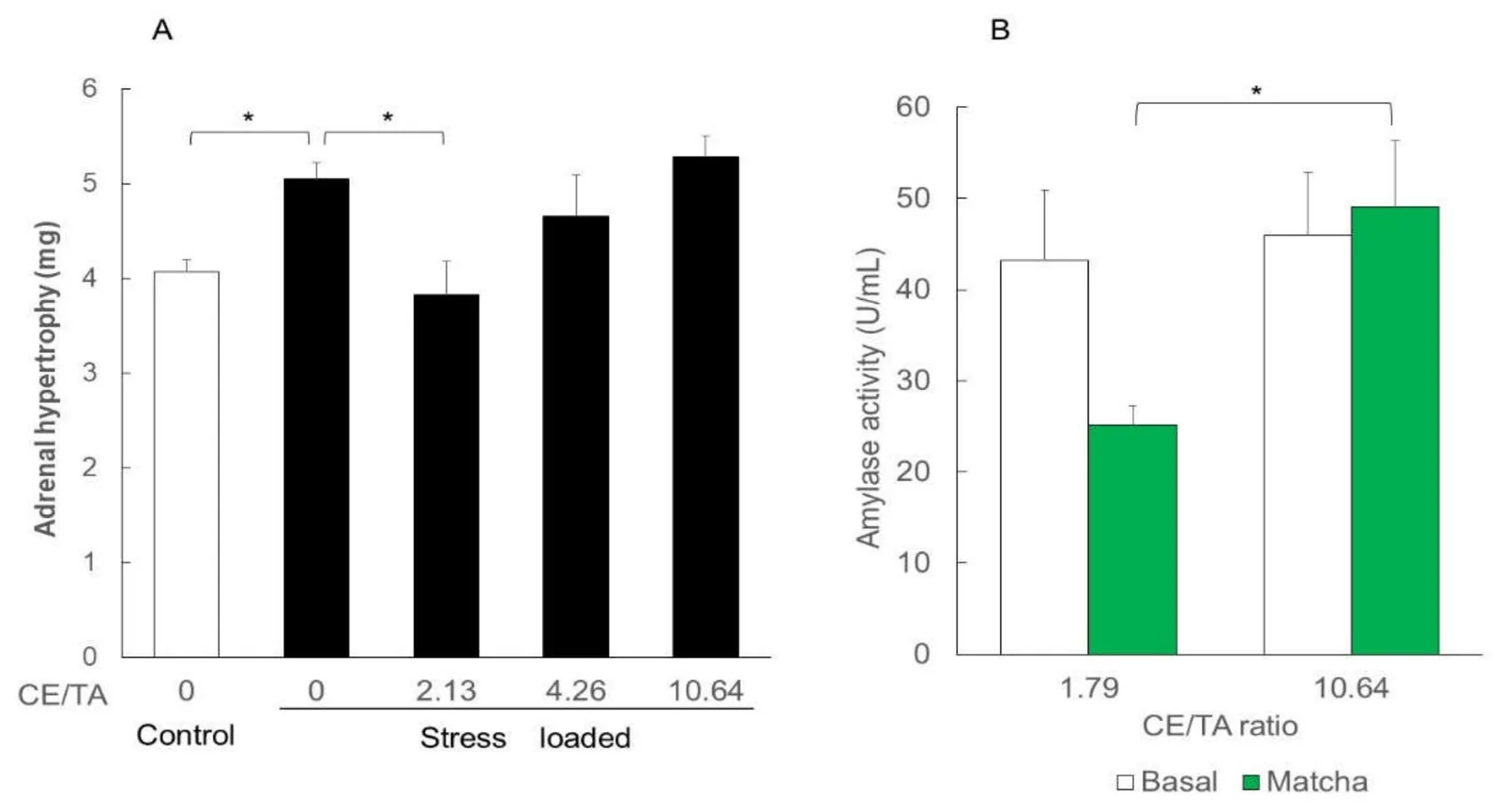
The relationship between the stress-reducing effect and the CE/TA ratio. Stress reduction in mice (**A**) and humans (**B**). The CE/TA ratio indicates the molar ratio of caffeine (C) and EGCG (E) to theanine (T) and arginine (A). In plot (**A**), purified theanine, arginine, caffeine, and EGCG were added to mouse feed at the following concentrations: CE/TA = 2.13 (T 50 μM, A 20 μM, C 86 μM, E 63 μM), CE/TA = 4.26 (T 12.5 μM, A 10 μM, C 86 μM, E 63 μM), and CE/TA = 10.64 (T 10 μM, A 4 μM, C 86 μM, E 63 μM). In stressed mice, stress reduction was evaluated by the suppression of adrenal gland hypertrophy. In plot (**B**), stress reduction was evaluated by the suppression of salivary amylase activity in humans (*, *p* < 0.05). The baseline values of salivary amylase activity before matcha consumption and the values for each matcha group under stressed conditions. Matcha (4.5 g) was mixed with flour (31.5 g), butter (26.1 g), sugar (13.5 g), and egg yolk (1.8 g), molded into three cookies, and then baked. Reproduced from Ref. [47].

In mice allowed to freely consume feed mixed with matcha (33–50 mg/kg)—which is proportionally equivalent to the human intake of matcha (2–3 g per 60 kg of body weight)—adrenal hypertrophy was significantly suppressed even under stressful conditions when the CE/TA ratio was 2 or less [33]. In contrast, no effect was observed with matcha when the CE/TA ratio was 3.2 or higher. When a similar experiment was conducted using feed supplemented with purified theanine, arginine, EGCG, and caffeine, a stress-reducing effect was observed when the CE/TA ratio was 2 or lower (Figure 5A) [47]. Similar effects were also observed when these compounds were administered via drinking water [41]. Furthermore, when theanine was administered in combination with arginine, EGCG, and caffeine, the amount of theanine required to demonstrate a stress-reducing effect was 1.8 mg/kg [41]. This concentration was higher than that required when theanine was administered alone.

[Human study] Stress levels were assessed using salivary amylase activity. When 3 g of matcha was suspended in water and ingested, salivary amylase activity decreased significantly compared to pre-ingestion levels for the matcha with a CE/TA ratio of 1.79, whereas no significant decrease was observed for the matcha with a CE/TA ratio of 10.64 [33]. Furthermore, daily consumption of 4.5 g of matcha in cookie form, with CE/TA ratios of 1.79 and 10.64, revealed that the group consuming matcha with a CE/TA ratio of 1.79 exhibited significantly lower salivary amylase activity compared with the group consuming matcha with a CE/TA ratio of 10.64 even under stressed conditions (Figure 5B) [47].

The CE/TA ratio in sencha was less than 3 in gyokuro and first-flush sencha tea but exceeded 3 in second- and autumn-flush teas (Table 3). This difference is attributed to the lower amino acid content and higher catechin content in second- and autumn-flush teas compared to first-flush tea.

When extrapolated to a 50–60 kg human based on animal study, an effective dose of theanine may correspond to approximately 100 mg [41], which could be estimated to be equivalent to an intake of at least 2.5 g of high-theanine matcha. Accordingly, matcha, sencha, and gyokuro with high theanine content are likely to exert stress-reducing effects, whereas green teas with low theanine content are unlikely to provide such effects.

### 5.2. Interaction Between Caffeine, Theanine, and EGCG

[In vitro study] A Caco-2 cell monolayer model was employed to investigate whether interactions between catechins and other tea components influence their absorption and efflux. The results demonstrated that incubation with 50 μM EGCG or EC for 3 h significantly increased the expression of multidrug resistance protein 2 and P-glycoprotein. In contrast, caffeine, theanine, glycine, and serine, which are all present in tea, suppressed the catechin-induced upregulation of these efflux transporters [48]. These findings suggest that the presence of caffeine or theanine may enhance the intestinal absorption of catechins. There is very little research on the interactions between these three components, and further studies are needed to confirm this.

### 5.3. Interaction Between Theanine and EGCG

#### 5.3.1. Interactive Effects on Neuronal Cells

Several epidemiological studies have suggested that green tea consumption may attenuate age-related cognitive decline [49,50,51]. The antioxidant properties and neuroprotective effects of catechins, particularly EGCG, are believed to contribute to this benefit [16,52,53]. In addition, reports have examined the effects of matcha consumption [23]. Given that matcha is rich in both catechins and theanine, these compounds may exert interactive effects on brain function. Although several studies suggest neuroprotective or cognitive benefits of green tea constituents, the overall evidence remains heterogeneous, and further well-controlled human studies are required.

[In vitro study] Xie et al. [54] investigated the effects of co-treatment with EGCG and theanine at equal molar concentrations (50 μM). Under β-amyloid-induced stress, neuronal damage leads to neurodegeneration through multiple mechanisms, including protein aggregation, inflammation, cell cycle dysregulation, and mitochondrial dysfunction. EGCG was shown to protect cellular membrane integrity and maintain intracellular protein homeostasis by suppressing amyloid-induced stress, reducing inflammation, and enhancing lipid and energy metabolism. In contrast, theanine exerted neuroprotective effects by enhancing cellular antioxidant capacity and preserving mitochondrial function and genetic material.

These findings suggest that the combined action of EGCG and theanine improves neuronal survival, promotes axonal outgrowth, and maintains the structural integrity of cellular proteins. However, there remain few studies on the relationship between the two, and further research is needed to determine the optimal ratio.

#### 5.3.2. Protective Effects on the Liver

Studies investigating the combined effects of EGCG and theanine have demonstrated that, compared with EGCG alone, the addition of theanine leads to reduced markers of hepatocellular damage, suppression of lipid peroxidation, and enhanced antioxidant enzyme activity [55,56]. These effects are thought to result from theanine-mediated regulation of amino acid metabolism, thereby mitigating EGCG-induced liver injury.

[Animal study] EGCG exhibits dose-dependent hepatoprotective effects at moderate doses (e.g., approximately 25–100 mg/kg in animal studies); however, higher doses (≥150 mg/kg) have been suggested to increase the risk of hepatotoxicity [57]. In contrast, theanine (e.g., 10–50 mg/kg) exhibits low toxicity when administered alone and may contribute to an expanded safety margin when co-administered with EGCG [55]. Furthermore, theanine has been reported to influence the pharmacokinetics of EGCG, potentially modulating its tissue distribution and metabolic fate [58].

In addition, the combination of EGCG and theanine has been shown to act interactively in the regulation of inflammatory signaling pathways (e.g., the NF-κB pathway) and apoptosis-related factors, demonstrating significant improvements even in models of non-alcoholic fatty liver disease [56]. These findings suggest that the two compounds do not solely exert antioxidant effects but also function complementarily at the level of cellular signaling. However, further research is needed regarding the ratio of these components.

#### 5.3.3. Protective Effects on Starch Digestibility

[In vitro study] Recent studies have demonstrated that EGCG and theanine exert synergistic inhibitory effects on starch digestion through coordinated interactions with digestive enzymes and starch structure. In vitro experiments showed that the combination of 1.6 mM theanine and 0.11 mM EGCG significantly enhanced α-glucosidase inhibition compared with either compound alone [59]. Mechanistically, both compounds non-covalently bind to key residues within the enzyme’s active site, inducing conformational changes that reduce catalytic efficiency.

Moreover, the combination of EGCG and theanine has been shown to reduce starch digestibility through multi-level structural modifications of starch [60]. These interactions decrease the proportion of rapidly digestible starch while promoting the formation of resistant starch. This interactive effect suggests that complex formation between starch and tea-derived components is a key determinant of digestibility. Collectively, these data suggest that EGCG and theanine act interactively at both the enzymatic and substrate levels, contributing to the suppression of postprandial blood glucose levels. It is necessary to verify whether these effects are confirmed in human trials and to determine the optimal ratio for maximum efficacy.

### 5.4. The Relationship Between Theanine and Caffeine

It has been reported that the combination of theanine and caffeine produces effects distinct from those observed with either compound alone, primarily influencing arousal, attention, and neural activity within the central nervous system.

[Animal study] In a study using rats, caffeine was shown to induce excitatory effects on electroencephalographic activity at doses of 5 μmol/kg (approximately 1 mg/kg) or higher. In contrast, theanine was found to suppress caffeine-induced excitation at a dose of 5 μmol/kg (approximately 0.8 mg/kg) [61]. This antagonistic effect was observed under conditions in which the molar ratio of the two compounds was comparable, suggesting that theanine modulates the central stimulatory effects of caffeine. Notably, low doses of theanine (1–2 μmol/kg) administered alone were found to induce mild excitation, indicating a dose-dependent biphasic effect [61].

[Human study] In a systematic review of the effects of green tea on human cognitive function, the combined influence of both theanine and caffeine was suggested [62], but cognitive evidence is heterogeneous. Human intervention studies have demonstrated improvements in attention and subjective alertness following the combined administration of theanine and caffeine. For example, the combination of 75 mg of caffeine and 50 mg of theanine has been shown to enhance performance in attention tasks, improve mood, and promote more stable cognitive performance compared with either compound administered alone [63]. Furthermore, studies examining combinations of theanine (3 mg/kg to 200 mg) and caffeine (3 mg/kg to 160 mg) have reported improvements in motor performance and inhibitory control, with superior effects observed relative to monotherapy, particularly in cognitive domains [64,65].

Analyses based on data from randomized controlled trials involving healthy participants have further evaluated the effects of combined theanine and caffeine on cognitive function, mood, and sleep. These findings suggest that both the combination of theanine and caffeine, as well as theanine alone, may confer beneficial effects on cognitive function and mood [66]. Collectively, the evidence indicates that theanine does not merely act as an antagonist to caffeine but also plays a regulatory role in modulating its stimulatory effects on the central nervous system. Since the ratio of theanine to caffeine is considered a key factor in optimizing these effects, further research is needed.

### 5.5. The Relationship Between Caffeine and EGCG

#### 5.5.1. Effects on Absorption Rate

[Human study] The absorption and metabolism of EGCG in the presence of caffeine have been examined with reference to the EGCG-to-caffeine ratios found in commercially available green tea beverages. Plasma EGCG measurements indicate that co-administration of 95 mg of EGCG with 40 mg of caffeine results in higher bioavailability compared to EGCG administered alone [67].

#### 5.5.2. Effects on the Nervous System

[In vitro study] Caffeine stimulates the central nervous system primarily through antagonism of adenosine A1 and A2A receptors, thereby promoting wakefulness and enhancing attention. EGCG, in contrast, is known to modulate neurotransmitter systems in addition to exerting antioxidant and neuroprotective effects [68,69]. Accordingly, EGCG may attenuate or modulate the stimulatory effects of caffeine on the central nervous system.

[Animal study] EGCG (15–30 mg/kg) has been shown to suppress the anxiogenic-like effects induced by caffeine (25 mg/kg) in mice [70]. This inhibitory effect is thought to be partly mediated through the regulation of dopaminergic transmission [70]. Furthermore, EGCG has been reported to attenuate caffeine-induced cardiovascular activation, including increases in blood pressure and heart rate, as well as elevations in circulating adrenaline and noradrenaline levels [71]. Future studies, including those with negative results, are needed to determine the optimal ratio of these two compounds.

#### 5.5.3. Anti-Obesity Effects

Both EGCG and caffeine modulate lipid metabolism; however, EGCG primarily exerts anti-obesity effects by inhibiting lipogenesis, whereas caffeine acts as an adjunct by increasing energy expenditure [72,73].

[Animal study] In rodent models of obesity, combined administration of EGCG (100 mg/kg/day) and caffeine (75 mg/kg/day) suppresses body weight gain and fat accumulation [74]. Even at lower doses (approximately 50 mg/kg EGCG plus 25 mg/kg caffeine), significant reductions in adiposity and hepatic fat have been observed, potentially involving alterations in gut microbiota composition and bile acid metabolism [75,76].

However, the interaction with caffeine appears to differ between EGCG as a single compound and mixtures of catechins [77], suggesting that EGCG alone may not fully account for the anti-obesity effects of catechins [78].

[Human study] Meta-analyses have generally concluded that the anti-obesity effects of green tea catechins are modest and subject to considerable inter-study heterogeneity. Habitual caffeine intake, ethnicity, and differences in study design may substantially influence the observed effects [79,80]. A systematic review on the effects of green tea supplementation on obesity noted that, while it may be associated with reductions in body weight and BMI in obese patients, it does not guarantee definitive effects [81]. Overall, although human studies support anti-obesity effects mediated by increased energy expenditure and enhanced fat oxidation, substantial variability exists among studies, and the effects are generally considered modest to limited [82].

## 6. Discussion

Matcha is typically prepared by suspending 2–3 g of powdered tea in hot water. A portion of catechins that do not fully dissolve in water are subsequently released into digestive fluids within the gastrointestinal tract [83], while components such as insoluble fibers are metabolized by the gut microbiota. As a result, a substantial proportion of matcha constituents are absorbed into the body. The composition of bioactive compounds ingested therefore varies depending on the type of matcha consumed. Matcha is generally rich in theanine and is thus expected to exert anti-stress effects. However, such effects are unlikely to be observed in processing-grade matcha or powdered tea products with low or negligible theanine content. Conversely, when the primary objective is to obtain the antioxidant effects of catechins or the health benefits associated with high catechin levels, processing-grade matcha or powdered tea may be more appropriate. These considerations highlight the importance of providing clear information regarding matcha quality.

In the case of sencha and gyokuro, bioactive compounds are consumed through the infusion prepared from tea leaves. The amounts of infused components vary depending on extraction conditions, such as water temperature and infusion time, which in turn influence the balance between umami and astringency. Accordingly, attention should be paid not only to leaf quality but also to infusion parameters. While most amino acids and caffeine are infused within the first few infusions, insoluble components and a portion of catechins remain in the leaves. However, sencha is typically consumed in multiple servings per day, allowing for adjustment of catechin and theanine intake.

We previously demonstrated that the CE/TA ratio serves as an indicator of stress-relieving effects under stress conditions [33]. Specifically, when the ratio of caffeine and catechins (stimulatory components) to theanine and arginine (inhibitory components) is three or less, the imbalance between excitation and inhibition induced by stress is effectively mitigated. In addition to the CE/TA ratio, a daily intake of theanine may be estimated to be necessary for humans [41].

It is currently understood that theanine binds strongly to glutamine transporters [84], thereby suppressing excessive production of glutamate, an excitatory neurotransmitter. Concurrently, theanine enhances the expression of neuronal PAS domain protein 4 (Npas4) [85], a transcription factor induced by neuronal activity that regulates the balance between excitatory and inhibitory neurons in the brain [86]. The dual action of low concentrations of theanine on both excitatory and inhibitory systems [61] is therefore considered to be mediated, at least in part, by Npas4.

Following oral ingestion, only 0.2–2% of EGCG is absorbed from the small intestine [87]. However, the presence of caffeine and theanine in green tea enhances its intestinal absorption [48,67]. EGCG that enters the bloodstream can cross the blood–brain barrier (BBB) and reach the brain in a rodent model [88]. Measurements using a BBB model system have shown that approximately 4% of EGCG permeates the BBB within 30 min, suggesting that roughly one-tenth of circulating EGCG reaches the brain.

Most EGCG, however, is metabolized by gut microbiota in the large intestine, producing 5-(3′,5′-dihydroxyphenyl)-γ-valerolactone and its sulfated derivatives as major metabolites [89,90]. These metabolites have also been shown to cross the BBB and promote neuronal differentiation [88].

Although EGCG has been reported to exert inhibitory or buffering effects on caffeine activity, this interaction may be altered in the presence of theanine, which modulates caffeine’s stimulatory effects. Furthermore, the influence of EGCG metabolites on excitatory and inhibitory neural processes remains an important subject for future investigation.

Extensive basic research has been conducted on catechins, caffeine, and amino acids, which are the principal functional components of green tea. Building on this foundation, recent studies have begun to elucidate the synergistic and antagonistic interactions among these compounds. Nevertheless, many questions remain unresolved, and further research is required to clarify these mechanisms. Increasing evidence suggests that green tea consumption contributes to the maintenance and promotion of both physical and mental health, particularly in relation to stress management and lifestyle-related conditions. At the same time, significant variation in the compositional profiles of different types of green tea has been documented. 

Several limitations should be acknowledged. First, many mechanistic studies discussed in this review were conducted using purified compounds, cultured cells, animal models, or non-Japanese green tea preparations. Direct extrapolation to all Japanese green tea products should therefore be made cautiously. Second, substantial heterogeneity exists among human studies regarding tea composition, dosage, habitual caffeine intake, and study design. Accordingly, further well-controlled studies using chemically characterized Japanese green tea preparations are required.

## 7. Conclusions

Green teas such as sencha, matcha, and gyokuro, which are derived from the leaves of the tea plant (*Camellia sinensis*), contain key components including catechins, caffeine, and theanine. However, the amounts and ratios of these components vary significantly among different types of green tea, resulting in differences in their functional effects.

For example, the balance of these components plays a crucial role in stress relief. Specifically, it has been suggested that the CE/TA ratio—defined as the molar ratio of the sum of caffeine (C) and EGCG (E), which exert stimulating effects, to the sum of theanine (T) and arginine (A), which exert inhibitory effects—should be less than 3. In addition, a daily intake of at least 100 mg of theanine might be considered necessary.

Because matcha involves the consumption of the entire tea leaf, it can be considered a direct and comprehensive method of green tea intake; however, the quality of the matcha itself is critical in determining its health benefits. In contrast, sencha and gyokuro are consumed as brewed infusions. The composition of their components can be adjusted through brewing conditions such as water temperature, allowing for flexible preparation methods in addition to the selection of tea leaves. Gyokuro, in particular, contains relatively high levels of theanine and is often regarded as one of the highest-quality green teas.

While numerous studies have examined the individual effects of green tea components, increasing attention has been paid to their interactions. These findings may provide useful guidance regarding the selection, timing, and method of green tea consumption to support both physical and mental health.

## Figures and Tables

**Table 1:** Types of Japanese green tea.

Green Tea	Type	Form	Shade-Grown	Rolling Process	Harvest Season	Theanine Content
Matcha (powdered Tencha)	Authentic (Ceremonial)	Powder	>20 days	-	First-flush	High
	Processed (Food-grade)	Powder	<20 days	-	Not determined	Low
Sencha	First	Needle	-	apply	First-flush	High
	Second	Needle	-	apply	Second-flush	Medium
	Autum/Bancha *	Needle	-	apply	Autum-flush or not determined	Low
	Powdered tea **	Powder	-	apply	Not determined	Low
Gyokuro		Needle	>20 days	apply	First-flush	High

*, Tea made from mature, hardened leaves; **, Finely ground sencha.

**Table 2:** Tea composition of matcha marketed in Japan and abroad.

Matcha	Japan		Abroad
Theanine Content	Higher	Lower	Middle
Theanine (mg/g)	41.4 ± 3.0	8.5 ± 0.6	8.7 ± 0.2
(μmol/g)	237 ± 17	49 ± 3	50 ± 1
Arginine (mg/g)	14.8 ± 3.8	1.4 ± 0.2	1.4 ± 0.4
(μmol/g)	85 ± 22	8 ± 1	8 ± 2
Glutamine	2.0 ± 1.1	0.7 ± 0.3	0.7 ± 0.1
Aspartic acid	9.5 ± 2.3	2.2 ± 0.7	2.4 ± 0.3
Glutamic acid	8.0 ± 2.4	2.4 ± 0.5	2.6 ± 0.2
Asparagine	3.7 ± 0.3	0.4 ± 0.2	0.5 ± 0.4
Serine	1.9 ± 0.2	0.6 ± 0.2	0.5 ± 0.2
GABA	0.2 ± 0.0	0.2 ± 0.1	0.2 ± 0.1
Caffeine (mg/g)	39.6 ± 4.2	33.4 ± 6.5	31.6 ± 5.1
(μmol/g)	204 ± 22	172 ± 34	163 ± 26
EGCG (mg/g)	57.3 ± 9.0	79.8 ± 12.7	75.8 ± 1.1
(μmol/g)	125 ± 20	174 ± 28	165 ± 2
EGC	10.2 ± 2.1	37.9 ± 8.2	32.7 ± 3.3
ECG	11.4 ± 2.1	14.4 ± 3.1	12.6 ± 2.4
EC	2.7 ± 0.7	8.3 ± 1.9	7.3 ± 1.5
CE/TA ratio	1.0 ± 0.1	6.2 ± 1.3	5.7 ± 0.1

These data (mean ± SD, *n* = 3) are reanalyzed data based on the results published in Reference [33] regarding 75 samples of matcha sold in Japan and 67 samples of matcha sold overseas in 2017. The column showing the amounts of each matcha component in μmol/g and CE/TA ratio are colored in gray.

**Table 3:** Tea components in sencha tea leaf.

Sencha		First Flush		Second Flush	Autumn Flush
		Earliest Stage	Middle Stage		(Bancha)
Theanine	mg/g	20.4	18.9	7.5	3.3
	μmol/g	117	109	428	187
Arginine	mg/g	5.6	4.7	0.8	0.3
	μmol/g	32	27	4	2
Glutamine	mg/g	4.5	3.7	1.5	0.6
Aspartic acid	mg/g	2.4	1.8	1.0	1.1
Glutamic acid	mg/g	3.0	2.6	1.5	1.3
Asparagine	mg/g	0.3	0.2	0.0	0.1
Serine	mg/g	0.8	0.7	0.4	0.2
Caffeine	mg/g	31.0	24.7	21.3	22.8
	μmol/g	160	127	110	117
EGCG	mg/g	67.5	62.2	75.1	82.4
	μmol/g	147	136	164	180
EGC	mg/g	20.1	25.9	47.2	52.2
ECG	mg/g	15.9	13.8	12.5	14.3
EC	mg/g	6.3	7.8	10.5	37.4
CE/TA ratio		2.1	1.9	5.8	14.6

This data is a synthesis of References [40,41]. For the first-flush tea, 18 pieces of green tea were collected from various regions across Japan in 2022, and nine of them were selected. These green teas were blended to create a test tea [40]. The second-flush sample was produced at the Mie Prefectural Agricultural Research Center for research purposes in 2023, as described in Ref. [41]. The autumn-flush sencha sample was commercially purchased from a supermarket in Mie Prefecture in 2023 [41]. The column showing the amounts of each tea component in μmol/g and CE/TA ratio are colored in gray.

**Table 4:** Tea components in sencha-infused solution.

Sencha		First Flush		Second Flush	Autumn Flush
		Earliest Stage	Middle Stage		(Bancha)
Leaf	g	6	6	5	5
Water Temperature	°C	70	90	80	80
Water volume	mL	170	260	200	200
Infusion time	min	1	1	1	1
Theanine	mg/L	256	218	94	21
	μmol/L	1470	1250	537	118
Arginine	mg/L	54	47	5	1
	μmol/L	311	271	27	5
Glutamine	mg/L	56	45	17	3
Aspartic acid	mg/L	41	27	13	7
Glutamic acid	mg/L	47	36	21	10
Asparagine	mg/L	5	2	0	1
Serine	mg/L	13	10	9	2
Caffeine	mg/L	305	333	238	212
	μmol/L	1570	1710	1220	1090
EGCG	mg/L	310	380	291	298
	μmol/L	676	830	635	651
EGC	mg/L	211	303	406	340
ECG	mg/L	68	87	48	46
EC	mg/L	68	80	92	73
CE/TA ratio		1.3	1.7	3.3	14.2

This data is a synthesis of References [40,41]. These values show the average based on data from three experiments for the first-flush tea. The data for the second-flush and autumn-flush teas are based on a single experiment [40,41]. The column showing the amounts of each tea component in μmol/L and CE/TA ratio are colored in gray.

**Table 5:** Components found in gyokuro tea leaves and their infusion.

Tea Components	Tea Leaf		Infused Solution *	
Theanine	31	mg/g	2807	mg/L
	177	μmol/g	16.1	mmol/L
Arginine	10	mg/g	722	mg/L
	56	μmol/g	4.1	mmol/L
Glutamine	5	mg/g	483	mg/L
Aspartic acid	6	mg/g	694	mg/L
Glutamic acid	4	mg/g	475	mg/L
Asparagine	2	mg/g	272	mg/L
Serine	1	mg/g	118	mg/L
Caffeine	35	mg/g	2261	mg/L
	182	μmol/g	11.6	mmol/L
EGCG	53	mg/g	1117	mg/L
	116	μmol/g	2.4	mmol/L
EGC	8	mg/g	478	mg/L
ECG	11	mg/g	233	mg/L
EC	3	mg/g	193	mg/L
CE/TA ratio	1.3		0.7	

Nine gyokuro teas were purchased (from three areas in Japan), and six were selected. These teas were blended for test material. The data for each infused solution represent the average of three examples. * Add 60 mL of water at 60 °C to 10 g of gyokuro tea leaves and steep for 2 min. This is a synthesis of the data presented in Reference [40]. The column showing the amounts of each tea component in μmol/g, mmol/L and CE/TA ratio are colored in gray.

## Data Availability

The original contributions presented in this study are included in the article. Further inquiries can be directed to the corresponding author.

## Abbreviations

The following abbreviations are used in this manuscript:

EGCG	Epigallocatechin gallate
EGC	Epigallocatechin
ECG	Epicatechin gallate
EC	Epicatechin
CE/TA ratio	Molar ratio of sum of caffeine and EGCG against sum of theanine and arginine
